# Assessment of frailty by paramedics using the clinical frailty scale - an inter-rater reliability and accuracy study

**DOI:** 10.1186/s12873-023-00875-x

**Published:** 2023-10-13

**Authors:** Christophe A. Fehlmann, Loric Stuby, Christophe Graf, Matthieu Genoud, Rebecca Rigney, Judah Goldstein, Debra Eagles, Laurent Suppan

**Affiliations:** 1 Division of Emergency Medicine, Department of Acute Medicine, Geneva University Hospitals, Rue Gabrielle-Perret-Gentil 4, Geneva, CH-1211 Switzerland; 2 School of Epidemiology and Public Health, University of Ottawa, Ottawa, ON K1G 5Z3 Canada; 3 Genève TEAM Ambulances, Emergency Medical Services, Geneva, CH-1201 Switzerland; 4 Department of rehabilitation and geriatrics, Geneva University Hospitals, Geneva, CH-1211 Switzerland; 5 National Ambulance Service, Westmeath, Ireland; 6 Dalhousie Department of Emergency Medicine, Division of EMS, Halifax, NS Canada; 7 Department of Emergency Medicine, University of Ottawa, Ottawa, ON Canada; 8 Clinical Epidemiology Program, Ottawa Hospital Research Institute, Ottawa, ON Canada

**Keywords:** Paramedics, Clinical frailty scale, Cfs, Reliability, Accuracy, Emergency medical services, Prehospital care, Triage system

## Abstract

**Background:**

Frailty assessment by paramedics in the prehospital setting is understudied. The goals of this study were to assess the inter-rater reliability and accuracy of frailty assessment by paramedics using the Clinical Frailty Scale (CFS).

**Methods:**

This was a cross-sectional study with paramedics exposed to 30 clinical vignettes created from real-life situations. There was no teaching intervention prior to the study and paramedics were only provided with the French version of the CFS (definitions and pictograms). The primary outcome was the inter-rater reliability of the assessment. The secondary outcome was the accuracy, compared with the expert-based assessment. Reliability was determined by calculating an intraclass correlation coefficient (ICC). Accuracy was assessed through a mixed effects logistic regression model. A sensitivity analysis was carried out by considering that an assessment was still accurate if the score differed from no more than 1 level.

**Results:**

A total of 56 paramedics completed the assessment. The overall assessment was found to have good inter-rater reliability (ICC = 0.87 [95%CI 0.81–0.93]). The overall accuracy was moderate at 60.6% (95%CI 54.9–66.1) when considering the full scale. It was however much higher (94.8% [95%CI 92.0–96.7] when close assessments were considered as accurate. The only factor associated with accurate assessment was field experience.

**Conclusion:**

The assessment of frailty by paramedics was reliable in this vignette-based study. However, the accuracy deserved to be improved. Future research should focus on the clinical impact of these results and on the association of prehospital frailty assessment with patient outcomes.

**Registration:**

This study was registered on the Open Science Framework registries (10.17605/OSF.IO/VDUZY).

**Supplementary Information:**

The online version contains supplementary material available at 10.1186/s12873-023-00875-x.

## Background

Frailty can be defined as state of vulnerability generated by the cumulative decline of several physiological systems. This decline results in a progressive depletion of patient reserves which can lead even minor stressor events to trigger disproportionate adverse effects [1–3]. Frailty prevalence among older patients in the Emergency Department (ED) is high, with up to two thirds of patients aged 65 years or older living with frailty [4]. Identification of frailty in the ED has been largely advocated [5]. It can however prove challenging as it requires acquiring data regarding the patient’s state prior to the current acute episode. Such data is not always readily available since patients are sometimes unable to communicate reliably, if at all. Therefore, obtaining relevant data in the prehospital phase could help ED clinicians take more appropriate decisions, such as discharge on scene or transport to a geriatric ED.

Prehospital assessment of frailty by paramedics, nurses or even physicians is currently understudied [6]. Since prehospital providers frequently respond at patients’ homes, they may have a more thorough understanding of the environment patients live in. Thus, prehospital assessment of frailty could be more accurate than ED assessment.

The main limitation of prehospital frailty assessment is the relative short time prehospital providers spend on site. Therefore, tools requiring either too much time or the availability of special equipment (such as the comprehensive geriatric assessment program) would not be a suitable option for these professionals. Simpler yet accurate tools should therefore be made available to prehospital providers. The Clinical Frailty Scale (CFS), whose score is based on clinical judgment, could be well suited for this task [7]. The CFS is a nine-point scale which extends from 1 (very fit) to 9 (terminally ill). It is considered easy to use, especially in busy clinical environments [8]. In the ED, it has been proven to be an accurate and reliable tool for predicting short-term and long-term mortality as well as an association with adverse events (initial admission rate, readmission, mortality) [9–11].

The use of the CFS in the prehospital environment has not been reported often and has scarcely been assessed [12, 13] Bernard et al. reported about Alternative Care Pathways (ACPs), a project aiming to reduce ED transport of patients with non-urgent needs who could be treated elsewhere [14]. In this cohort, patients had a median CFS of 6. Two other studies showed that frailty prevalence was around 60% [15,16]. More recently, authors showed that use of the CFS by paramedics was feasible [17, 18]. However, little information about the training, reliability, or accuracy of the CFS was reported in those studies. It is nevertheless essential to demonstrate that the use of the Clinical Frailty Scale by paramedics is reliable and accurate if one wants to use frailty to guide decision making in the prehospital environment.

There is therefore a knowledge gap regarding the use of the CFS in the prehospital setting. The goals of this study were to assess the inter-rater reliability and accuracy of frailty assessment using the CFS and to identify factors associated with accurate CFS assessment among paramedics.

## Methods

### Design and setting

This was a closed web-based cross-sectional study carried out on Swiss paramedics working in Geneva, Switzerland. It was designed according to the Checklist for Reporting Results of Internet E-Surveys (CHERRIES) and is reported accordingly (Appendix I) [19]. This study was registered on the Open Science Framework [20].

Swiss paramedics follow a three-year education program which includes theoretical lectures, simulation workshops, and field internships [21]. There are seven ambulances companies in Geneva, five of which are privately owned and operated, while the two others are state-run. Together, they take care of more than 35’000 patients per year [22]. In Geneva, there is currently no frailty screening by paramedics. However, physicians working in the prehospital medical mobile unit perform frailty screening using the Clinical Frailty Scale. There is however no formal screening in the Emergency Department, contrarily to many hospitals from the German-speaking part of Switzerland. Since this study design does not fall within the scope of the Swiss Federal Act on Research Involving Human Beings, the need for a formal IRB approval was waived by the president of the regional ethics committee (“clarification of responsibility”, Req-2022-00921).

### Web-based platform and study procedure

A specific web-based platform was developed using the Joomla! 4.2 content management system (Open Source Matters, New York, USA) and thoroughly tested by four investigators prior to study inception. The AcyMailing 7.9 component (Acyba, Lyon, France) was used to send individual invitation email to all the paramedics working in Geneva between February and March 2023. Their email addresses were obtained through the companies’ chief ambulance officers, all of whom endorsed this study. To promote participation, all chief medical officers agreed to award continuous education credits to the paramedics who completed the study. This was the only incentive and participation was entirely voluntary. Invitation reminders were sent twice at 14-day intervals.

The invitation email contained information regarding the study’s aim and design, including the time required to complete it. It was signed by the principal investigator (CF), and a generic email address was provided to allow paramedics to ask further questions to the study team. The participants who chose to click on the link to the study platform were directed to the platform’s main page where they were reminded of the study’s aim, design, and data protection procedures. Since paramedics often follow continuous medical education interventions while at work, it was considered that they could be interrupted at any time during the study and were therefore asked to create unique accounts. To avoid attrition, the registration form was kept as short as possible: participants were only asked to provide an e-mail address, enter a password, and provide electronic informed consent. A Completely Automated Public Turing test to tell Computers and Humans Apart (CAPTCHA v2, Google LLC, Mountain View, USA) was also used to avoid the creation of fake accounts. The registration process was managed using the Membership Pro 3 component (Joomdonation, Hanoi, Vietnam).

Joomla’s access control list was used to manage the study sequence. Before accessing the clinical vignettes, participants were asked to answer a first questionnaire designed to gather demographic data. This questionnaire was created using Shondalai’s Community Survey 5.9 component (Bulasikku Technologies, Hyderabad, India). After completing this step, the paramedics accessed the 30 clinical vignettes in random order. This was managed using Shondalai’s Community Quiz 6.3 component (Bulasikku Technologies, Hyderabad, India), and participants were able to leave the platform at any time and to resume the study path at will without data loss. It was not possible to skip from one vignette to another and participants were required to provide an answer before moving on the next vignette. For each vignette, participants were asked to assess the frailty level, using the CFS. No formal training was provided, but for each vignette, the official CFS (French version) was displayed along with the CFS pictograms. A certificate was automatically awarded once the 30 clinical vignettes were completed.

All data was stored in an encrypted MySQL-compatible database (MariaDB 10.3, MariaDB Foundation, Delaware, USA) hosted on a Swiss server (Kreativ Media GmbH, Zurich, Switzerland). Admin Tools Professional 7 (Akeeba Ltd, Nicosia, Cyprus) and RS Firewall 3 (RSJoomla!, Constanta, Romania) were used to secure the platform from external intrusion.

### Clinical vignettes

Thirty clinical vignettes were created by the main author (CF), based on real-life patients brought to the ED by paramedics (names were changed). The main characteristics of the patients described in the vignettes are displayed in Table 1. All the data deemed necessary to assess the CFS were provided with no need to search for specific information. There was no possibility to gather further information. The vignettes were reviewed and tested by three of the co-authors (CG, LSu, LSt). The detailed vignettes (in French, with English translation) are available as supplementary material (Appendix II).

**Table 1:** Characteristics of vignettes’ patients

Patients	N = 30
Patient’s gender – n (%)	
Women	14 (46.7)
Men	16 (53.3)
Patient’s age (years) – median (IQR)	79 (74–86)
Patient’s age (years) – n (%)	
65–75	9 (30.0)
76–80	8 (26.7)
81–86	6 (20.0)
> 86	7 (23.0)
Living in a nursing home	
No	26 (86.7)
Yes	4 (13.3)
Clinical Frailty Scale – n (%)	
1	3 (10.0)
2	3 (10.0)
3	4 (13.3)
4	5 (16.7)
5	4 (13.3)
6	3 (10.0)
7	3 (10.0)
8	3 (10.0)
9	2 (6.7)

A reference CFS was defined for each vignette by a panel of multidisciplinary experts (one research paramedic, one board-certified geriatrician, and one physician certified in emergency medicine and specialized in prehospital emergency medicine). Each of them assessed the CFS independently. Disagreements were mostly caused by unclear or ambiguous sentences. They were resolved by consensus and led to appropriate scenario modifications.

### Outcomes

The primary outcome was the inter-rater reliability of frailty assessment. The secondary outcome were the accuracy of the assessment compared with the reference CFS, using specific definitions (inaccurate, under-assessment and over-assessment), and factors associated with accurate CFS assessment. An assessment was considered accurate if the paramedic assigned the same CFS level as the reference. Overassessment and underassessment were defined with regard to the reference CFS level.

### Statistical analysis

Continuous variables were presented by their median and interquartile ranges, and categorical variables by their frequency and relative proportions. To measure the inter-rater reliability in frailty assessment among the participants, an intraclass correlation coefficient (ICC) with its 95% confidence interval (CI) was calculated using a two-way random effects model (absolute agreement). The ICC was interpreted in line with prior publications: values less than 0.5, between 0.5 and 0.75, between 0.75 and 0.9, and greater than 0.90 were considered indicative of poor, moderate, good, and excellent reliability, respectively [23].

For the accuracy, we first reported the proportion of correct assessment of each rater and for each vignette, with their 95% CI. Then, considering the fact that the observations are not truly independent (same paramedics, same scenarios), we reported the overall accuracy and its 95%CI. They were estimated using a mixed effects logistic regression model with crossed random effects on the intercept. We also reported the absolute differences between the reference CFS and the one assessed by paramedics. We performed one post-hoc sensitivity analysis, by considering that an assessment was still accurate if the score differed from no more than 1 level.

We then realised an exploratory analysis to assess the factors associated with the accuracy of CFS assessment by performing a generalized linear mixed model using a logit function and a vignette-random effects on the intercept. The model was adjusted for the following prespecified variables: gender of the paramedic, experience of the paramedic in years, patient’s gender and age, and place of living (long-term care facility or not). These variables were chosen based on previous knowledge of their influence on frailty assessment. As patient age and field experience did not respect the assumption of the linearity of the log-odds, categories were created and cut-off points were chosen using quartiles. For each variable, we reported an adjusted odds ratio (aOR) with its 95% CI. All analyses were performed using Stata version 17 (Stata Corp., College Station, Texas, USA). Statistical significance was defined as a P value < 0.05 (two sided).

### Sample size calculation

The number of clinical vignettes was fixed (N = 30). Data were crossed as the same 30 clinical vignettes were allocated to each paramedic. Only the vignette order randomly varied from one paramedic to another. Using the formula provided by Bonett, [24] and expecting an ICC of 0.80, the number of paramedics needed for a precision of +/- 0.1 was of 22. A sample of 50 paramedics was nevertheless planned to allow multivariable analyses without a risk of overfitting. More participants were accepted as there was no risk for them, and because it could prevent overfitting even further in the multivariable model.

## Results

**Fig. 1:**
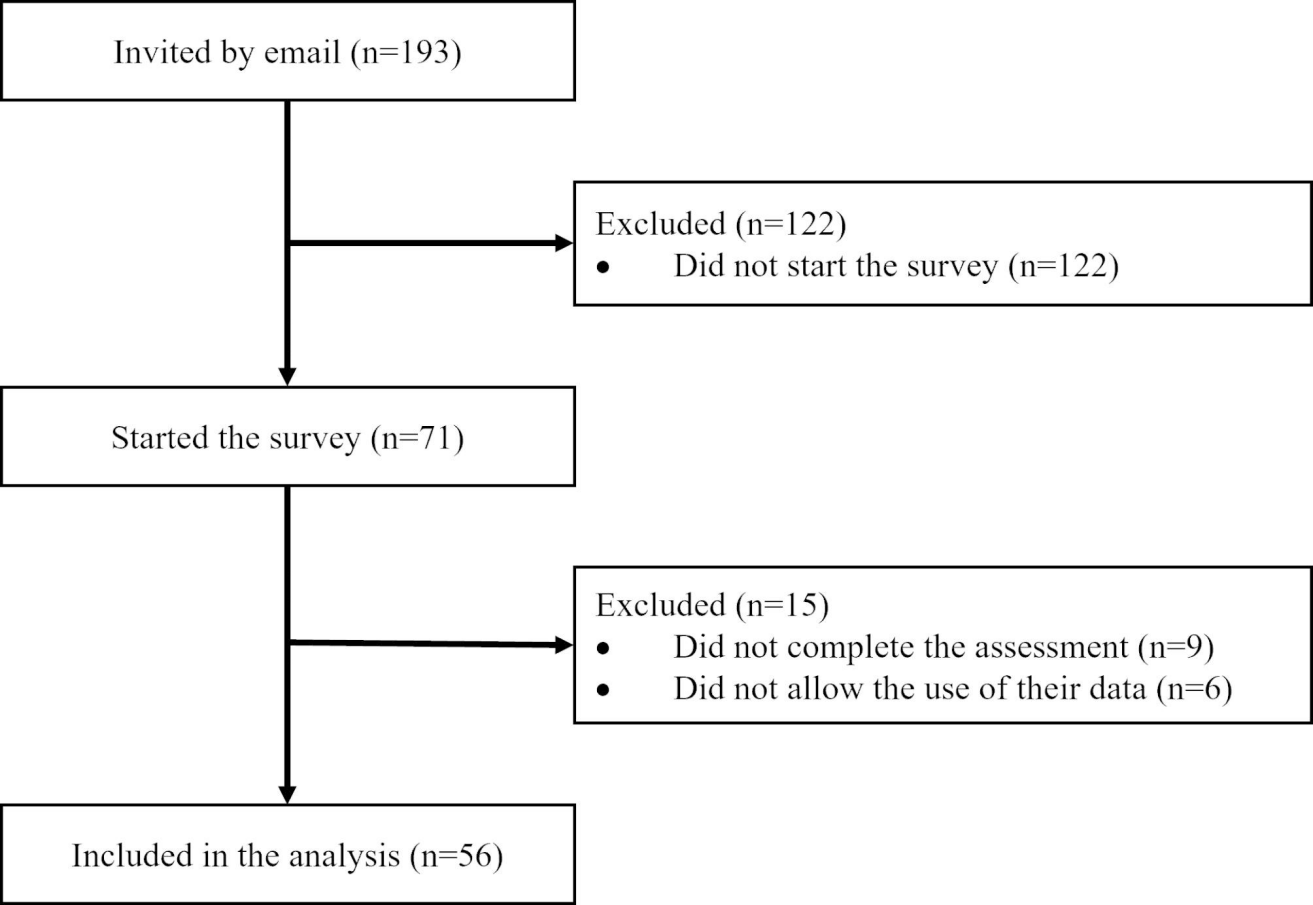
Flowchart of the study

Of all invited paramedics (n = 193), 56 (29%) completed the assessment and met eligibility. They were thus included in the final analysis (Fig. 1) and their characteristics can be seen in Table 2. Thirty-two of them (57.1%) were men. The median age was 31.5 years (IQR 28.0–37.5), with a median field experience of 7 years (IQR 3–12). Before this study, only 4 paramedics (7.1%) had heard about the CFS, and none of them had ever used it in clinical practice.

**Table 2:** Characteristics of the paramedics

Paramedics	N = 56
Gender of paramedics – n (%)	
Women	23 (41.1)
Men	32 (57.1)
Other	1 (1.8)
Paramedic’s experience (years) – median (IQR)	7 (3–12)
Paramedic’s experience (years) – n (%)	
0–3	16 (28.6)
4–7	13 (23.2)
8–12	14 (25.0)
> 12	13 (23.2)
Previous knowledge of Clinical Frailty Scale – n (%)	
No	52 (92.9)
Yes	4 (7.1)

**Fig. 2:**
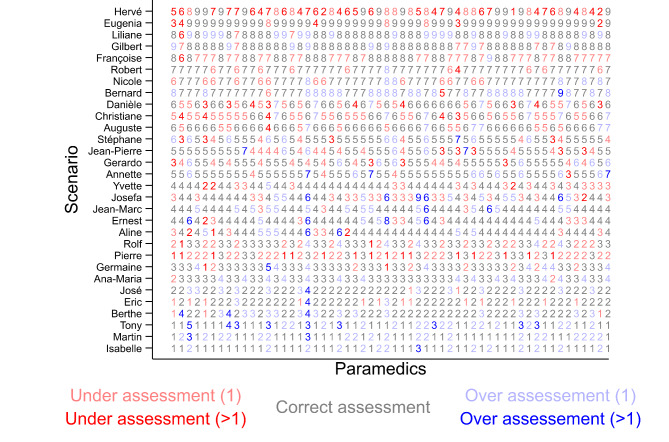
Answers of each paramedic to each vignette

Regarding the overall inter-rater reliability, the ICC was 0.87 (95%CI 0.81–0.93). It was similar between men (0.86 [95%CI 0.80–0.92]) and women (0.87 [95%CI 0.81–0.93]) paramedics and also similar between men (0.87 [95%CI 0.78–0.95]) and women (0.87 [95%CI 0.78–0.95]) patients. The agreement rate by vignette varied between 23.2% (one vignette, “Hervé”) and 85.7% (one vignette, “Eugenia”) (Fig. 2). Figure 2 shows the answers of each paramedic to each vignette.

**Fig. 3:**
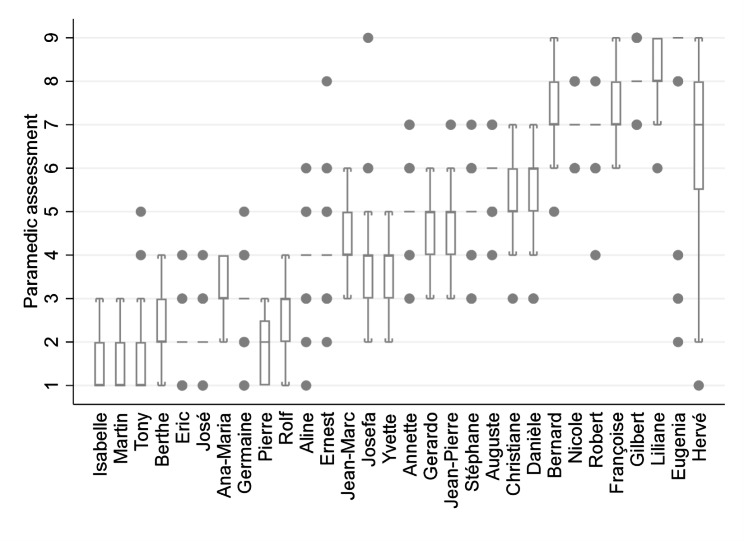
Agreement by vignettes

Regarding the accuracy, 1’008 (60.0%) assessments were correct, 288 (17.1%) were over-assessments and 384 (22.9%) were under-assessments. Among the 672 inaccurate assessments, 538 (80.1%) deviated by only one level from the reference (Fig. 3). The overall accuracy was 60.6% (95%CI 54.9–66.1); the median correct assessment rate was 64.3% (IQR 53.4–69.6) by vignette and 61.7% (IQR 51.7–66.7) by paramedic. Our sensitivity analysis showed higher accuracy: the overall accuracy was 94.8% (95%CI 92.0–96.7) when close assessments (deviations of no more than one level) were considered as accurate.

Only field experience was associated with accurate assessment, with paramedics who had between 4 and 7 years of field experience providing less accurate assessments (OR = 0.66, 95%CI 0.50–0.88) (Table 3).

**Table 3:** Predictors of correct assessment

	OR	95%CI
Gender of paramedics		
Women	Ref.	
Men	1.01	0.82–1.24
Other	1.27	0.56–2.85
Paramedic’s experience in prehospital care (years)		
0–3	Ref.	
4–7	0.66	0.50–0.88
8–12	0.78	0.58–1.03
> 12	0.93	0.70–1.25
Patient’s gender		
Women	Ref.	
Men	0.91	0.57–1.45
Patient’s age (years)		
65–75	Ref.	
76–80	0.91	0.50–1.69
81–86	0.69	0.36–1.32
> 86	1.30	0.70–2.40
Living in a nursing home		
No	Ref.	
Yes	1.27	0.64–2.50

## Discussion

In this study based on clinical vignettes, the assessment of frailty by paramedics using the Clinical Frailty Scale had an excellent inter-rater reliability albeit a moderate accuracy.

### 1° reliability

Many studies assessed the reliability of the CFS, mostly based on the assessment of unique real patients by two different raters [25, 26]. Some studies have however assessed reliability with designs similar to ours. In a study by Nissen et al., 40 health care providers rated 15 clinical case scenarios with an good reliability (ICC = 0.85) [27]. In a small study comparing the assessment of seven vignettes by 124 care providers also showed a good agreement, with median CFS scores varying by a maximum of only one point [28]. Our study confirms that CFS assessment by paramedics is highly reproductible, even with a no prior training.

### 2° accuracy

In our study, when compared to a reference defined by a multidisciplinary team of experts in their field, the accuracy of CFS assessment by untrained paramedics assessment was not optimal, with an accurate assessment rate around 60%. In a study looking at the effect of training on the accuracy of the assessment by registered nurses, the median overall agreement was 55.8% [29]. When comparing assessment by medical students to expert assessment, Kaeppeli et al. did not find a perfect agreement either (Kappa = 0.74) [30]. The weak accuracy of the CFS assessment might be explained by the high numbers levels at disposition, some of which with differences that might not be perceptible by non-expert. This hypothesis is strengthened by our sensitivity analysys which showed a much higher accuracy when close assessments were considered as accurate. A 1-point discrepancy in the CFS score might indeed be considered as a negligible difference. The design of our study might also explain this suboptimal accuracy, as vignettes might have been possibly too vague in some situations. While training nurses experienced in the use of the Clinical Frailty Scale does not seem to improve the accuracy, we believe that an initial training of unexperienced assessor could improve the accuracy of the assessment.

We also identified field experience as a potential predictor of accuracy: paramedics with 4 to 7 years of professional experience were less likely to give an accurate assessment compared to less experienced professionals. However, this association seems to have a J shape, and it is difficult to distinguish whether it is a true better accuracy for inexperienced paramedics, it results from newly graduate paramedics being more attentive in the reading of the vignette or it is a type I error.

### 3° limitation and strengths

This study was based on vignettes and not on a simulator. Therefore, participants did not have to actively collect the information needed to assess frailty. It could potentially overestimate the results of the study, as all variables needed for the assessment were presented right away. On the contrary, in real life paramedics might use the visual representation of the situation (patient general appearance, place of living, etc.) in their assessment, which could improve their assessment. Another limitation was the recruitment, which was based purely on volunteers, even if a high rate of paramedics did participate to the study. As participant could have a special interest for geriatric patients, their performance could be better than that of their less interested colleagues. The main strength of this study is the comprehensive statistical analysis, which carried out using the CFS both as a continuous variable and as a binary variable.

### 4° clinical implication

Some practical implications can be mentioned. Based on this study, it could be beneficial to train paramedics, before implementing a systematic regular screening in prehospital, to enhance the accuracy. While some training materials exist, none was specifically developed for paramedics, and training modules should be adapted to this specific population.

The use of the CFS in the prehospital field could help in identifying the older patients living with frailty and at highest risk of adverse outcomes, and therefore require more specialized care to improve their outcomes. Early identification of vulnerability, particularly among older patients who are frequently transported to the hospital, is needed [31, 32]. A better triage of those patients could also help to reduce ED workload, either by helping paramedics to orientate them to geriatrics wards, or to release them on-site and thus contribute to decrease ED overload [33]. From a patient perspective, early identification of frailty level by paramedics might optimise the triage process on arrival in the ED, improve communication between clinicians, patients and families, and also facilitate transitions in care, by activating discharge planning staff prior to in-hospital assessments.

Moreover, the use of the CFS in the context of out-of-hospital cardiac arrest could help make difficult clinical decisions and predict outcomes after return of spontaneous circulation. Indeed, frailty is associated with survival and with cognitive and functional status after cardiac arrest [34–36].

### 5° research implication

Several questions remain to be answered. The use of the CFS during real prehospital interventions should be studied to assess its feasibility and to identify barriers and difficulties paramedics might encounter. Such a study could also help assess the actual accuracy of CFS assessment by paramedics, by comparing their assessment to that of a specialist geriatrician. Then, it could be useful to study the association between prehospital frailty assessed by CFS with outcomes such as patients’ disposition (by paramedics but also after ED stay) and mortality [16].

## Conclusion

The assessment of frailty by paramedics using the Clinical Frailty Scale was reliable in this vignette-based study. The accuracy nevertheless deserved to be improved. Future research should focus on the clinical impact of these results and on the association of prehospital frailty assessment with patient outcomes.

### Electronic supplementary material

Below is the link to the electronic supplementary material.

Appendix I ? CHERRIES Checklist. Appendix II ? Clinical vignettes with English translation.

## Data Availability

The dataset supporting the conclusions of this article is available in the Open Science Framework repository [10.17605/OSF.IO/4ETPV].

## Declarations

### Previous study

The main author is also the first author of a study (under review) with a very similar design and identical statistical analysis process that was performed to assess a paediatric triage scale. It might explain the similar structure and method description between both articles.

## References

[1] Clegg A, Young J, Iliffe S, Rikkert MO, Rockwood K (2013). Frailty in elderly people. Lancet.

[2] Rockwood K, Song X, MacKnight C (2005). A global clinical measure of fitness and frailty in elderly people. Can Med Assoc J.

[3] WHO Clinical Consortium on Health Ageing. Geneva, Switzerland: World Health Organization, 2017.

[4] Choutko-Joaquim S, Tacchini-Jacquier N, Pralong D, Alessio G, Verloo H (2019). Associations between Frailty and Delirium among older patients admitted to an Emergency Department. Dement Geriatric Cogn Disorders Extra.

[5] Eagles D, Ellis B, Melady D, Frailty (2020). A key concept to improve older person care. CJEM.

[6] Goldstein J, Andrew MK, Travers A (2012). Frailty in older adults using Pre-hospital Care and the Emergency Department: a narrative review. Can Geriatr J.

[7] Rockwood K, Song X, MacKnight C (2005). A global clinical measure of fitness and frailty in elderly people. CMAJ.

[8] Lewis ET, Dent E, Alkhouri H (2019). Which frailty scale for patients admitted via Emergency Department? A cohort study. Arch Gerontol Geriatr.

[9] Lee JH, Park YS, Kim MJ (2022). Clinical Frailty Scale as a predictor of short-term mortality: a systematic review and meta-analysis of studies on diagnostic test accuracy. Acad Emerg Med.

[10] Rueegg M, Nissen SK, Brabrand M (2022). The clinical frailty scale predicts 1-year mortality in emergency department patients aged 65 years and older. Acad Emerg Med.

[11] Elliott A, Taub N, Banerjee J et al. Does the clinical Frailty Scale at Triage Predict Outcomes from Emergency Care for Older People? Annals of emergency medicine 2021;77(6):620–7. 10.1016/j.annemergmed.2020.09.006. 10.1016/j.annemergmed.2020.09.006 33328147

[12] Fehlmann CA, Nickel CH, Cino E, Al-Najjar Z, Langlois N, Eagles D (2022). Frailty assessment in emergency medicine using the clinical Frailty Scale: a scoping review. Intern Emerg Med.

[13] Alshibani A, Alharbi M, Conroy S (2023). Frailty Identification in Prehospital Care: a scoping review of the literature. Open Access Emerg Med.

[14] Bernard P, Corcoran G, Kenna L et al. Is pathfinder a safe alternative to the emergency department for older patients? An observational analysis. Age and ageing 2021(0375655, 2xr). 10.1093/ageing/afab095. 10.1093/ageing/afab095 34107008

[15] Charlton K, Sinclair DR, Hanratty B, Burrow E, Stow D. Measuring frailty and its association with key outcomes in the ambulance setting: a cross sectional observational study. BMC Geriatr. 2022;22(1). 10.1186/s12877-022-03633-z. 10.1186/s12877-022-03633-z PMC9721042 36471316

[16] Sinclair DR, Charlton K, Stow D, Burrow E, Hanratty B (2023). Care Home Residency and its Association with Ambulance Service workload. J Am Med Dir Assoc.

[17] Charlton K, Sinclair DR, Hanratty B, Burrow E, Stow D (2022). Measuring frailty and its association with key outcomes in the ambulance setting: a cross sectional observational study. BMC Geriatr.

[18] Morton S, Gough C (2023). Can you assess the clinical Frailty Scale in the HEMS setting? A feasibility study. Emerg Med J.

[19] Eysenbach G (2004). Improving the quality of web surveys: the Checklist for reporting results of internet E-Surveys (CHERRIES). J Med Internet Res.

[20] Open Science Framework. (https://osf.io/).

[21] Schmutz T, Guechi Y, Denereaz S, et al. Paramedics in Switzerland: a mature Profession. Int J Environ Res Public Health. 2022;19(14). 10.3390/ijerph19148429. 10.3390/ijerph19148429 PMC9316220 35886281

[22] Chappuis VN, Deham H, Cottet P, et al. Emergency physician’s dispatch by a paramedic-staffed emergency medical communication centre: sensitivity, specificity and search for a reference standard. Scand J Trauma Resusc Emerg Med. 2021;29(1). 10.1186/s13049-021-00844-y. 10.1186/s13049-021-00844-y PMC7871575 33563301

[23] Koo TK, Li MY (2016). A Guideline of selecting and reporting Intraclass correlation coefficients for Reliability Research. J Chiropr Med.

[24] Bonett DG (2002). Sample size requirements for estimating intraclass correlations with desired precision. Stat Med.

[25] Lo AX, Heinemann AW, Gray E (2020). Inter-rater reliability of clinical Frailty Scores for older patients in the Emergency Department. Acad Emerg Med.

[26] Shears M, Takaoka A, Rochwerg B (2018). Assessing frailty in the intensive care unit: a reliability and validity study. J Crit Care.

[27] Nissen SK, Fournaise A, Lauridsen JT, et al. Cross-sectoral inter-rater reliability of the clinical frailty scale – a danish translation and validation study. BMC Geriatr. 2020;20(1). 10.1186/s12877-020-01850-y. 10.1186/s12877-020-01850-y PMC7640648 33143651

[28] Young RL, Smithard DG, The Clinical Frailty Scale (2020). Do Staff Agree? Geriatrics.

[29] Leblanc A, Diab N, Backman C (2022). Development and assessment of an educational intervention to improve the recognition of frailty on an acute care respiratory ward. BMJ Open Quality.

[30] Kaeppeli T, Rueegg M, Dreher-Hummel T et al. Validation of the clinical Frailty Scale for Prediction of thirty-day mortality in the Emergency Department. Annals of emergency medicine 2020. 10.1016/j.annemergmed.2020.03.028. 10.1016/j.annemergmed.2020.03.028 32336486

[31] Goldstein J, Mcvey J, Ackroyd-Stolarz S (2016). The role of Emergency Medical Services in Geriatrics: bridging the gap between primary and Acute Care. CJEM.

[32] Vuilleumier S, Fiorentino A, Denereaz S, Spichiger T (2021). Identification of new demands regarding prehospital care based on 35,188 missions in 2018. BMC Emerg Med.

[33] Leduc S, Cantor Z, Kelly P, Thiruganasambandamoorthy V, Wells G, Vaillancourt C (2021). The safety and effectiveness of On-Site paramedic and Allied Health Treatment Interventions Targeting the reduction of Emergency Department visits by Long-Term Care Patients: systematic review. Prehospital Emerg care: Official J Natl Association EMS Physicians Natl Association State EMS Dir.

[34] Mowbray FI, Turcotte L, Strum RP et al. Prognostic Association between Frailty and Post-Arrest Health Outcomes in patients receiving Home Care: a Population-Based Retrospective Cohort Study. Resuscitation 2023:109766. 10.1016/j.resuscitation.2023.109766. 10.1016/j.resuscitation.2023.109766 36931455

[35] Yamamoto R, Tamura T, Haiden A et al. Frailty and neurologic outcomes of patients resuscitated from nontraumatic out-of-hospital cardiac arrest: a prospective observational study. Annals of emergency medicine 2023. 10.1016/j.annemergmed.2023.02.009. 10.1016/j.annemergmed.2023.02.009 36964008

[36] Goldstein J, Rockwood K, Lee JS. Pre-arrest frailty and implications for cardiac arrest care. Resuscitation 2023:109793. 10.1016/j.resuscitation.2023.109793. 10.1016/j.resuscitation.2023.109793 37044355

