# Supplementary material for: Assessment of frailty by paramedics using the clinical frailty scale - an inter-rater reliability and accuracy study
Source: BMC Emerg Med. 2023 Oct 13;23:121. doi: 10.1186/s12873-023-00875-x (PMC10576312; doi:10.1186/s12873-023-00875-x)
Supplement: Supplementary file 1 — Appendix I ? CHERRIES Checklist. Appendix II ? Clinical vignettes with English translation. [file 12873_2023_875_MOESM1_ESM.docx]

# Supplemental material

## Appendix I – CHERRIES Checklist

| ***Item Category*** | ***Checklist Item*** | ***Explanation*** | ***Comment*** |
| --- | --- | --- | --- |
| **Design** | Describe survey design | Describe target population, sample frame. Is the sample a convenience sample? (In “open” surveys this is most likely.) | In "Design and setting": *All participants were Swiss paramedics working in Geneva, Switzerland.* In "Statistical analysis and sample size": *A sample of 50 paramedics was nevertheless planned to allow multivariable analyses without a risk of overfitting.* |
| **IRB (Institutional Review Board) approval and informed consent process** | IRB approval | Mention whether the study has been approved by an IRB. | In "Design and setting": *Since this study design does not fall within the scope of the Swiss Federal Act on Research Involving Human Beings, the need for a formal IRB approval was waived by the president of the regional ethics committee* |
|  | Informed consent | Describe the informed consent process. Where were the participants told the length of time of the survey, which data were stored and where and for how long, who the investigator was, and the purpose of the study? | In "Web-based platform and study procedure": *To avoid attrition, the registration form was kept as short as possible: participants were only asked to provide an e-mail address, enter a password, and provide electronic informed consent.* & *The participants who chose to click on the link to the study platform were directed to the platform’s main page where they were reminded of the study’s aim, design, and data protection procedures* |
|  | Data protection | If any personal information was collected or stored, describe what mechanisms were used to protect unauthorized access. | In "Web-based platform and study procedure": *All data was stored in an encrypted MySQL-compatible database (MariaDB 10.3, MariaDB Foundation, Delaware, USA) hosted on a Swiss server (Kreativ Media GmbH, Zurich, Switzerland). Admin Tools Professional 7 (Akeeba Ltd, Nicosia, Cyprus) and RS Firewall 3 (RSJoomla!, Constanta, Romania) were used to secure the platform from external intrusion.* |
| **Development and pre-testing** | Development and testing | State how the survey was developed, including whether the usability and technical functionality of the electronic questionnaire had been tested before fielding the questionnaire. | In "Web-based platform and study procedure": *A specific web-based platform was developed using the Joomla! 4.2 content management system (Open Source Matters, New York, USA) and thoroughly tested by 4 investigators prior to study inception.* & in "Clinical scenarios": *Thirty scenarios were created by the main author (CF), based on real-life patients brought to the ED by paramedics.* |
| **Recruitment process and description of the sample having access to the questionnaire** | Open survey versus closed survey | An “open survey” is a survey open for each visitor of a site, while a closed survey is only open to a sample which the investigator knows (password-protected survey). | In "Design and setting": *This was a closed web-based cross-sectional study* |
|  | Contact mode | Indicate whether or not the initial contact with the potential participants was made on the Internet. (Investigators may also send out questionnaires by mail and allow for Web-based data entry.) | In "Web-based platform and study procedure": *The AcyMailing 7.9 component (Acyba, Lyon, France) was used to send individual email to all the paramedics working in Geneva. Their email addresses were obtained through the companies’ chief ambulance officers, all of whom endorsed this study.* |
|  | Advertising the survey | How/where was the survey announced or advertised? Some examples are offline media (newspapers), or online (mailing lists – If yes, which ones?) or banner ads (Where were these banner ads posted and what did they look like?). It is important to know the wording of the announcement as it will heavily influence who chooses to participate. Ideally the survey announcement should be published as an appendix. | See above |
| **Survey administration** | Web/E-mail | State the type of e-survey (eg, one posted on a Web site, or one sent out through e-mail). If it is an e-mail survey, were the responses entered manually into a database, or was there an automatic method for capturing responses? | In "Web-based platform and study procedure": *the paramedics accessed the 30 clinical scenarios in random order. This was managed using Shondalai’s Community Quiz 6.3 component (Bulasikku Technologies, Hyderabad, India),* |
|  | Context | Describe the Web site (for mailing list/newsgroup) in which the survey was posted. What is the Web site about, who is visiting it, what are visitors normally looking for? Discuss to what degree the content of the Web site could pre-select the sample or influence the results. For example, a survey about vaccination on a anti-immunization Web site will have different results from a Web survey conducted on a government Web site | In "Web-based platform and study procedure": *A specific web-based platform was developed using the Joomla! 4.2 content management system (Open Source Matters, New York, USA)* |
|  | Mandatory/voluntary | Was it a mandatory survey to be filled in by every visitor who wanted to enter the Web site, or was it a voluntary survey? | In "Web-based platform and study procedure": *This was the only incentive and participation was entirely voluntary.* |
|  | Incentives | Were any incentives offered (eg, monetary, prizes, or non-monetary incentives such as an offer to provide the survey results)? | In "Web-based platform and study procedure": *To promote participation, all chief medical officers agreed to award continuous education credits to the paramedics who completed the study. This was the only incentive and participation was entirely voluntary.* |
|  | Time/Date | In what timeframe were the data collected? | In "Web-based platform and study procedure": *The AcyMailing 7.9 component (Acyba, Lyon, France) was used to send individual invitation email to all the paramedics working in Geneva between September 2022 and December 2022.* |
|  | Randomization of items or questionnaires | To prevent biases items can be randomized or alternated. | In "Web-based platform and study procedure": *After completing this step, the paramedics accessed the 30 clinical scenarios in random order.* |
|  | Adaptive questioning | Use adaptive questioning (certain items, or only conditionally displayed based on responses to other items) to reduce number and complexity of the questions. | No adaptive questioning used (implicit) |
|  | Number of Items | What was the number of questionnaire items per page? The number of items is an important factor for the completion rate. | Since this was not a standard survey, this item does not apply to this particular study |
|  | Number of screens (pages) | Over how many pages was the questionnaire distributed? The number of items is an important factor for the completion rate. | Since this was not a standard survey, this item does not apply to this particular study |
|  | Completeness check | It is technically possible to do consistency or completeness checks before the questionnaire is submitted. Was this done, and if “yes”, how (usually JAVAScript)? An alternative is to check for completeness after the questionnaire has been submitted (and highlight mandatory items). If this has been done, it should be reported. All items should provide a non-response option such as “not applicable” or “rather not say”, and selection of one response option should be enforced. | In "Web-based platform and study procedure": *It was not possible to skip from one scenario to another and participants were required to provide an answer before moving to the next scenario.* |
|  | Review step | State whether respondents were able to review and change their answers (eg, through a Back button or a Review step which displays a summary of the responses and asks the respondents if they are correct). | Since this was not a standard survey, this item does not apply to this particular study |
| **Response rates** | Unique site visitor | If you provide view rates or participation rates, you need to define how you determined a unique visitor. There are different techniques available, based on IP addresses or cookies or both. | In "Web-based platform and study procedure": *Since paramedics often follow continuous medical education interventions while at work, it was considered that they could be interrupted at any time during the study and were therefore asked to create unique accounts. To avoid attrition, the registration form was kept as short as possible: participants were only asked to provide an e-mail address, enter a password, and provide electronic informed consent. A Completely Automated Public Turing test to tell Computers and Humans Apart (CAPTCHA v2, Google LLC, Mountain View, USA) was also used to avoid the creation of fake accounts. The registration process was managed using the Membership Pro 3 component (Joomdonation, Hanoi, Vietnam).* |
|  | View rate (Ratio of unique survey visitors/unique site visitors) | Requires counting unique visitors to the first page of the survey, divided by the number of unique site visitors (not page views!). It is not unusual to have view rates of less than 0.1 % if the survey is voluntary. | Since this was not a standard survey, this item does not apply to this particular study |
|  | Participation rate (Ratio of unique visitors who agreed to participate/unique first survey page visitors) | Count the unique number of people who filled in the first survey page (or agreed to participate, for example by checking a checkbox), divided by visitors who visit the first page of the survey (or the informed consents page, if present). This can also be called “recruitment” rate. | See flowchart |
|  | Completion rate (Ratio of users who finished the survey/users who agreed to participate) | The number of people submitting the last questionnaire page, divided by the number of people who agreed to participate (or submitted the first survey page). This is only relevant if there is a separate “informed consent” page or if the survey goes over several pages. This is a measure for attrition. Note that “completion” can involve leaving questionnaire items blank. This is not a measure for how completely questionnaires were filled in. (If you need a measure for this, use the word “completeness rate”.) | See flowchart |
| **Preventing multiple entries from the same individual** | Cookies used | Indicate whether cookies were used to assign a unique user identifier to each client computer. If so, mention the page on which the cookie was set and read, and how long the cookie was valid. Were duplicate entries avoided by preventing users access to the survey twice; or were duplicate database entries having the same user ID eliminated before analysis? In the latter case, which entries were kept for analysis (eg, the first entry or the most recent)? | See "Unique site visitor" |
|  | IP check | Indicate whether the IP address of the client computer was used to identify potential duplicate entries from the same user. If so, mention the period of time for which no two entries from the same IP address were allowed (eg, 24 hours). Were duplicate entries avoided by preventing users with the same IP address access to the survey twice; or were duplicate database entries having the same IP address within a given period of time eliminated before analysis? If the latter, which entries were kept for analysis (eg, the first entry or the most recent)? | Since this was not a standard survey, this item does not apply to this particular study |
|  | Log file analysis | Indicate whether other techniques to analyze the log file for identification of multiple entries were used. If so, please describe. |  |
|  | Registration | In “closed” (non-open) surveys, users need to login first and it is easier to prevent duplicate entries from the same user. Describe how this was done. For example, was the survey never displayed a second time once the user had filled it in, or was the username stored together with the survey results and later eliminated? If the latter, which entries were kept for analysis (eg, the first entry or the most recent)? | See "Unique site visitor" |
| **Analysis** | Handling of incomplete questionnaires | Were only completed questionnaires analyzed? Were questionnaires which terminated early (where, for example, users did not go through all questionnaire pages) also analyzed? | See flowchart |
|  | Questionnaires submitted with an atypical timestamp | Some investigators may measure the time people needed to fill in a questionnaire and exclude questionnaires that were submitted too soon. Specify the timeframe that was used as a cut-off point, and describe how this point was determined. | Since this was not a standard survey, this item does not apply to this particular study |
|  | Statistical correction | Indicate whether any methods such as weighting of items or propensity scores have been used to adjust for the non-representative sample; if so, please describe the methods. | See "Statistical analysis" section in the methods |

## Appendix II – Clinical vignettes with English translation

| Original version | English translation |
| --- | --- |
| Aline, 92 ans. Vit dans un IEPA*, au centre-ville. Elle est connue pour une polyarthrite rhumatoïde, traitée, mais qui progresse. Bien qu’elle commence à avoir des difficultés à sortir de chez elle pour faire ses courses, car les douleurs sont assez importantes, elle se force encore à les faire 3x/semaine. Elle fait encore son ménage et ses payements seule, mais se demande si cela va durer longtemps. | Aline is a 92-year-old woman who lives in a IEPA* in the city center. She is known to have rheumatoid arthritis, which is being treated but is progressing. Although she is starting to have difficulty leaving her home to do her shopping, as the pain is quite significant, she still forces herself to do so three times a week. She still cleans and manages her payments on her own, but wonders how much longer she will be able to continue doing so. |
| Ana-Maria, 66 ans, ancienne employée de commerce. Vit avec son mari à domicile. Elle est connue pour un antécédent de by-pass gastrique, et un diabète bien contrôlé sous metformine. Elle ne sort pas beaucoup de chez elle, préférant passer son temps à cuisiner, activité moins fatigante pour elle, et qu’elle apprécie particulièrement. | Ana-Maria is a 66-year-old former business employee who lives at home with her husband. She is known to have a history of gastric bypass surgery and well-controlled diabetes under metformin. She does not go out much, preferring to spend her time cooking, which is less tiring for her and something she particularly enjoys. |
| Annette, 72 ans. Elle vit dans un D2*, depuis 18 mois, car cela devenait impossible de rester à la maison. Elle prend plusieurs médicaments, surtout pour sa dépression et ses anxiétés. Elle va de temps en temps faire des courses, mais uniquement si sa fille est avec elle pour l’aider à payer. Elle a une infirmière à domicile 1x/j, principalement pour la prise de médicaments. Ses repas lui sont également livrés à domicile, et une femme de ménage passe 1x/ semaine. | Annette is a 72-year-old woman who has been living in a IEPA* for 18 months because it had become impossible for her to stay at home. She takes several medications, mainly for her depression and anxiety. She occasionally goes shopping, but only if her daughter is with her to help pay. She has a home nurse who visits her once a day, mainly for medication management. Her meals are also delivered to her home, and a cleaning lady comes once a week. |
| Auguste, 88 ans. Ancien gros fumeur, il a eu un cancer du poumon il y a 10 ans, a première vue sans récidive. Il vit aujourd’hui en EMS*, car ses enfants n’arrivaient plus à l’aider suffisamment quand il était encore dans son appartement. Il se déplace seul avec un déambulateur, mais doit être aidé le matin pour enfiler son pantalon. | Auguste is an 88-year-old man who used to smoke heavily. He had lung cancer 10 years ago, which appears to be without recurrence. He now lives in a nursing home because his children were no longer able to provide enough help when he was still in his apartment. He moves around on his own with a walker, but needs assistance in the morning to put on his pants. |
| Bernard, 77 ans. Il est connu pour une cardiopathie rythmique avec un pacemaker, un diabète, une obésité sévère, une insuffisance artérielle avec amputation mi-jambe à droite, une insuffisance rénale, et plusieurs AVC avec séquelles. Il vit en EMS*, et nécessite de l’aide pour toutes les activités du quotidien, y compris pour manger. | Bernard is a 77-year-old man known to have a rhythm heart disease with a pacemaker, diabetes, severe obesity, arterial insufficiency with mid-leg amputation on the right, kidney failure, and several strokes with sequelae. He lives in a nursing home and requires assistance with all activities of daily living, including eating. |
| Berthe, 94 ans. Vit dans un IEPA*, depuis le décès de son mari il y a 6 mois, car elle ne supportait plus l’isolement. Elle est connue pour une cataracte débutante et une constipation chronique, pour laquelle elle prend des laxatifs. Bien qu’elle ait du plaisir à inviter ses petits-enfants au restaurant, elle regrette de ne plus avoir autant d'interactions sociales qu'auparavant. Elle passe beaucoup de temps à jouer au bridge avec ses amies. Elle fait encore un peu de ski de fond l’hiver, s’il y a assez de neige. | Berthe, 94 years old, lives in a IEPA* since her husband's death 6 months ago because she could no longer tolerate isolation. She has early-stage cataracts and chronic constipation, for which she takes laxatives. Although she enjoys inviting her grandchildren to restaurants, she regrets not having as much social interaction as before. She spends a lot of time playing bridge with her friends. She still does a little cross-country skiing in the winter if there is enough snow. |
| Christiane, 79 ans. Elle est connue pour un diabète, une hypertension, et une insuffisance rénale chronique, dialysée 3x/semaine. Elle vit seule à la maison, avec une gouvernante présente uniquement la journée. Celle-ci l'aide dans la majorité des tâches du domicile, et l'accompagne aux séances de dialyse. Christiane occupe la plus grande partie de son temps par la lecture. | Christiane is a 79-year-old woman known to have diabetes, hypertension, and chronic kidney failure, requiring dialysis 3 times a week. She lives alone at home, with a housekeeper present only during the day. The housekeeper helps her with most of the household tasks and accompanies her to dialysis sessions. Christiane spends most of her time reading. |
| Danièle, 74 ans. Elle vit à domicile, avec son mari. Elle est connue pour une sclérose en plaque, qui évolue très lentement mais depuis de nombreuses années. Son mari est très présent, et il effectue la majorité des tâches du foyer, aidé par leur enfant. Une aide-soignante passe 3x/sem pour l’aide à la douche, que son mari ne peut effectuer. En dehors de cela, Danièle est relativement autonome. | Danièle is a 74-year-old woman who lives at home with her husband. She is known to have multiple sclerosis, which has been progressing very slowly for many years. Her husband is very present and does most of the household tasks, with help from their child. A caregiver comes 3 times a week to assist with showering, which her husband cannot do. Other than that, Danièle is relatively independent. |
| Eric, 78 ans. Vit dans sa maison sur la rive droite, en campagne, avec son épouse. Il est connu pour un cancer de la prostate, opéré il y a 5 ans. Ancien jardinier, il a continué à pratiquer son activité, en s’occupant de son jardin mais également de ceux des voisins. Il fait quelques randonnées l’automne, surtout pour récolter des champignons. | Eric is a 78-year-old man living in his house on the right bank (*of the Rhône, Translator's note*), in the countryside, with his wife. He had prostate cancer surgery 5 years ago. A former gardener, he continued to practice his activity by taking care of his own garden and those of his neighbors. He goes on a few hikes in the fall, especially to collect mushrooms. |
| Ernest, 74 ans, connu pour un antécédent de dissection aortique avec bioprothèse, et deux prothèses de hanches. Veuf, il vit dans un 3 pièce au centre-ville. Jusqu'au mois passé, il allait régulièrement faire de la peinture les jeudi après-midi au centre des ainés de son quartier, mais depuis quelques semaines il ne trouve plus vraiment la force de s'y rendre. | Ernest, 74 years old, known for a history of aortic dissection with bioprosthesis and two hip prostheses. A widower, he lives in a 3-room apartment in the city center. Until last month, he regularly went to the senior center in his neighborhood to paint on Thursday afternoons, but for the past few weeks he hasn't really found the strength to go there. |
| Eugenia, 93 ans. Connue pour un cancer de l’estomac, avec des métastases hépatiques, pulmonaires et cérébrales, le tout diagnostiqué il y a 1 mois. Après discussion avec sa famille, elle avait décidé de ne pas entreprendre de traitement, et de profiter du temps qui lui restait avec ses petits-enfants, son chat et son jardin. | Eugenia, 93 years old. Known for stomach cancer, with hepatic, pulmonary, and cerebral metastases, all diagnosed 1 month ago. After discussing with her family, she decided not to undergo treatment and to enjoy the time left with her grandchildren, cat, and garden. |
| Françoise, 104 ans. Centenaire, avec comme seule pathologie majeure une polyarthrite rhumatoïde très sévère, avec d’importantes déformations et de très grosses douleurs. Elle vit chez elle, avec un important encadrement (gouvernante 24/7) et est alitée en permanence. | Françoise, 104 years old. A centenarian, with only one major pathology, which is severe rheumatoid arthritis with significant deformities and very severe pain. She lives at home with significant support (24/7 caregiver) and is bedridden permanently. |
| Gerardo, 79 ans. Il est connu pour un asthme modéré, avec également une cardiopathie ischémique et valvulaire. Il vit seul, dans un appartement au centre-ville. Il adore la lecture, et est un grand amateur de poésie anglaise. Il s’occupe lui-même de ses factures, mais ne sort plus de chez lui à cause de son asthme. Son fils lui fait les courses 3 fois par semaines, et une femme de ménage passe 2x/semaine. | Gerardo, 79 years old. He is known for moderate asthma, as well as ischemic and valvular heart disease. He lives alone in an apartment in the city center. He loves reading and is a great fan of English poetry. He takes care of his bills himself but no longer goes out due to his asthma. His son does the shopping for him 3 times a week, and a cleaning lady comes twice a week. |
| Germaine, 85 ans. Vit à domicile, avec son mari, qui est connu pour une démence progressive depuis quelques années. Elle s’occupe de la majorité des tâches administratives y compris les payements, mais préfère que la pharmacie prépare les médicaments de son mari. Elle gère seule son antihypertenseur. Elle sort uniquement pour faire les courses, car elle s’inquiète beaucoup pour son mari. | Germaine, 85 years old. Lives at home with her husband, who has been known to have progressive dementia for a few years. She takes care of most of the administrative tasks, including payments, but prefers the pharmacy to prepare her husband's medications. She manages her antihypertensive medication alone. She only goes out to do the shopping because she is very worried about her husband. |
| Liliane, 86 ans. Elle est connue pour un cancer du poumon depuis 10 ans, avec plusieurs lignes de chimiothérapie, sans succès. Depuis 6 mois, les traitements ont été stoppés, afin de favoriser son confort, et Liliane ne sort plus de son lit. Elle vit à domicile, avec son mari qui l’aide du mieux qu’il peut. Une aide-soignante vient 1x/j pour la toilette au lit, et l’aider à s’habiller. | Liliane, 86 years old. She is known for lung cancer for 10 years, with several lines of chemotherapy without success. For the last 6 months, treatments have been stopped to promote her comfort, and Liliane no longer gets out of her bed. She lives at home with her husband who helps her as best he can. A nursing assistant comes once a day for bed baths and to help her get dressed. |
| Josefa, 85 ans. Elle vit chez sa fille, depuis la mort de son mari il y a 5 ans. Elle est connue pour une fibrillation auriculaire, une leucémie myéloïde chronique, un diabète et une insuffisance rénale chronique, pour lequel elle prend de nombreux traitements. Comme sa fille travaille beaucoup, Josefa s’occupe de la maison (nettoyage, lessive) et prépare le repas du soir, en fonction des courses faites par ses petits-enfants car elle ne sort plus vraiment. | Josefa, 85 years old. She lives with her daughter since her husband's death 5 years ago. She is known for atrial fibrillation, chronic myeloid leukemia, diabetes, and chronic kidney failure, for which she takes numerous medications. As her daughter works a lot, Josefa takes care of the house (cleaning, laundry) and prepares dinner based on the groceries purchased by her grandchildren as she doesn't really go out anymore. |
| Gilbert, 78 ans. Il est connu pour une démence de type vasculaire depuis quelques années, très sévère, avec une perte d’autonomie complète, justifiant son admission il y a 18 mois dans un EMS* spécialisé. Il a de nombreux troubles de la déglutition, avec de multiples épisodes de pneumonie d’aspiration ces 3 derniers mois. Il est récemment sorti de l’hôpital, avec des directives anticipées demandant de favoriser le confort lors du prochain épisode et d’éviter le transfert à l’hôpital. | Gilbert, 78 years old. He is known to have vascular dementia for several years, very severe, with complete loss of autonomy, justifying his admission 18 months ago in a specialized nursing home. He has many swallowing disorders, with multiple episodes of aspiration pneumonia in the last 3 months. He has recently been discharged from the hospital, with advance directives asking to promote comfort during the next episode and to avoid transfer to the hospital. |
| Hervé, 84 ans. Opéré il y a 6 mois d’une fracture du fémur suite à une chute à vélo, qui s’est compliqué d’une infection de prothèse, avec une longue hospitalisation, et de multiples cures d’antibiothérapie. Après 3 reprises au bloc, Hervé a décidé qu’il ne voulait plus rester à l’hôpital et préférait rentrer chez lui, malgré les conséquences de l’arrêt d’une antibiothérapie iv. | Hervé, 84 years old. He underwent surgery 6 months ago for a femur fracture following a bike accident, which was complicated by a prosthesis infection, with a long hospital stay and multiple courses of antibiotics. After 3 surgeries, Hervé decided he did not want to stay in the hospital anymore and preferred to go home, despite the consequences of stopping an IV antibiotic therapy. |
| Isabelle, 77 ans, retraitée. Opérée il y a 1 an d’une fracture du col du fémur, survenant suite à une chute à la montagne. Elle prend du Calcium et de la vitamine D, pour prévenir une nouvelle fracture. Elle fait partie d’un groupe de marche, avec lesquelles elle pratique des randonnées à la journée dans les alpes environnantes, 2-3x/semaines. | Isabelle, 77 years old, retired. She underwent surgery 1 year ago for a fractured neck of femur following a fall in the mountains. She takes calcium and vitamin D to prevent a new fracture. She is part of a walking group and practices day hikes in the surrounding Alps 2-3 times a week. |
| Jean-Marc, 85 ans. Il vit dans un appartement en ville, avec son compagnon. Il est connu pour deux antécédents d’infarctus, et de multiples épisodes de décompensation cardiaque. Il aimait beaucoup marcher dans la vieille-ville, mais depuis 12 à 18 mois cela est devenu plus difficile, car il est très vite essoufflé. Il passe donc beaucoup de temps à la maison, à faire des mots croisés. | Jean-Marc, 85 years old. He lives in an apartment in the city with his partner. He has a history of two heart attacks and multiple episodes of heart failure. He used to enjoy walking in the old town, but for the past 12 to 18 months, it has become more difficult for him as he quickly becomes breathless. He spends a lot of time at home doing crosswords. |
| Jean-Pierre, 86 ans. Vit en appartement protégé* avec son épouse, avec un passage quotidien de l'IMAD* pour ses traitements (Aspirine, Co-Candesartan, Beloc-Zok, Simvastatine, Cipralex). Relativement stable sur le plan somatique, il présente un état dépressif chronique, qui a engendré une perte d'autonomie progressive ces dernières années. | Jean-Pierre, 86 years old. Lives in a protected apartment* with his wife, with a daily visit from the home care service for his medications (aspirin, candesartan cilexetil + hydrochlorothiazide, metoprolol, simvastatin, escitalopram). He is relatively stable on the physical level, but he has a chronic depressive state, which has led to a progressive loss of autonomy in recent years. |
| José, 74 ans. Connu pour une BPCO débutante, répondant bien aux aérosols. Il vit au centre-ville, avec son épouse et 3 chats. Son épouse est très malade, en raison d’un cancer du sein, raison pour laquelle l’IMAD* l’aide dans les soins qu’il procure à son épouse. Il va régulièrement à la piscine de Carouge, l’été, pour faire quelques longueurs et se changer la tête. | José, 74 years old. Known for early-stage COPD, responding well to aerosols. He lives in the city center with his wife and 3 cats. His wife is very ill due to breast cancer, which is why the home care service helps him with the care he provides for his wife. He regularly goes to the swimming pool in Carouge during the summer to swim a few laps and clear his mind. |
| Martin, 68 ans, avocat encore en pratique. Connu pour un infarctus il y a 5 ans, raison pour laquelle il prend de l’aspirine et du Beloc. Il joue au tennis avec son fils 2-3x/semaine, à midi, et fait des randonnées à ski durant les week-ends d’hiver. | Martin, 68 years old, is a practicing lawyer. He is known to have had a heart attack 5 years ago, which is why he takes aspirin and metoprolol. He plays tennis with his son 2-3 times a week at noon and goes skiing on weekends during the winter. |
| Nicole, 70 ans. BPCO très sévère, importante cachexie sur malnutrition. Vit en EMS* depuis 4 ans, suite à l’introduction de l’oxygène, qu’elle n’arrivait pas à gérer au domicile. Ses enfants et ses petits-enfants lui rendent souvent visite, ou l’emmène au restaurant d’à côté, ce qu’elle apprécie particulièrement. Très dépendante en raison de sa dyspnée, elle nécessite de l’aide pour la douche et l’habillage, et ne se déplace qu’en chaise roulante, avec sa bouteille d’oxygène. | Nicole, 70 years old. Very severe COPD, significant cachexia due to malnutrition. She has been living in a nursing home for 4 years, since the introduction of oxygen, which she was unable to manage at home. Her children and grandchildren often visit her or take her to the nearby restaurant, which she particularly enjoys. Due to her dyspnea, she is highly dependent and requires assistance with showering and dressing, and can only move around in a wheelchair, with her oxygen tank. |
| Pierre, 66 ans, ancien médecin aux HUG*. Connu pour de l'arthrose, majoritairement au niveau dorsal, bien contrôlée sous AINS et paracétamol. Vit à domicile avec son conjoint, avec lequel il passe la majorité des week-ends à la montagne, à se balader dans le village. | Pierre, 66 years old, former doctor at the HUG*. Known for osteoarthritis, mostly in the dorsal region, well-controlled with NSAIDs and paracetamol. Lives at home with his partner, with whom he spends most weekends in the mountains, walking around the village. |
| Robert, 93 ans. Ancien sportif de haut niveau, Robert est connu pour une hypertension et un diabète. Il y a 8 mois, il a présenté un très important AVC, avec une hémiplégie quasi-complète à droite. Par conséquence, il ne peut plus se déplacer ou s’habiller seul, et nécessite également de l’aide pour la douche. Il a cependant encore énormément de plaisir à jouer aux cartes avec son épouse. | Robert, 93 years old. Former high-level athlete, Robert is known for hypertension and diabetes. Eight months ago, he had a very severe stroke, resulting in almost complete right-side hemiplegia. As a result, he can no longer move or dress himself, and also needs help with showering. However, he still enjoys playing cards with his wife a lot. |
| Rolf, 79 ans. Ancien policier à la retraite. Vit à domicile, seul. Il est connu pour une fibrillation auriculaire, pour laquelle il prend du Sintrom et du Beloc, sans problème particulier. S’il s’ennuie un peu en semaine restant majoritairement chez lui, il se réjouit des week-ends, car il s’occupe de sa dernière petite-fille de 3 ans, avec laquelle il passe quelques heures au parc. | Rolf, 79 years old. Retired police officer, living alone at home. He is known for atrial fibrillation, for which he takes acenocoumarol and metoprolol without any particular problems. While he may feel a bit bored during the weekdays, mostly staying at home, he looks forward to the weekends, when he takes care of his youngest granddaughter who is 3 years old. They spend a few hours together at the park. |
| Stéphane, 78 ans. Il vit avec son épouse, à domicile. Depuis quelques années maintenant, il présente des troubles cognitifs, qui évoluent plutôt lentement. Il passe beaucoup de temps devant la télévision, mais également au téléphone avec sa petite-fille qui vit au Canada. Il prépare souvent le repas avec son épouse, mais c’est elle qui fait les courses et lui prépare ses médicaments, car il n’est plus capable de le faire tout seul. | Stéphane, 78 years old. He lives with his wife at home. For a few years now, he has been experiencing cognitive impairments that are evolving slowly. He spends a lot of time watching television, but also on the phone with his granddaughter who lives in Canada. He often helps his wife prepare meals, but she is the one who goes grocery shopping and prepares his medication because he is no longer able to do it himself. |
| Tony, 93 ans, retraité. Veuf depuis 5 ans suite au décès de sa femme. Il vit dans son appartement, au bord du lac. Hospitalisé 3 jours l’hiver passé en raison d’une infection à COVID. Le retour à domicile s’est bien passé. Il passe la majorité de ses journées au parc, à marcher avec son chien, ou sur le lac, seul sur voilier. | Tony, 93 years old, retired. Widowed for 5 years following the death of his wife. He lives in his apartment by the lake. Hospitalized for 3 days last winter due to a COVID infection. The return home went well. He spends most of his days at the park, walking with his dog, or on the lake, sailing alone. |
| Yvette, 68 ans. Elle est connue pour un état dépressif chronique et un léger surpoids. Elle vit avec son mari, dans une maison à la campagne. Elle conduit de temps en temps, pour se rendre sur la tombe de son fils, décédé dans un accident il y a 5 ans. Elle aimerait bien reprendre la peinture, mais se sent assez fatiguée. | Yvette, 68 years old. She is known for chronic depressive state and slight overweight. She lives with her husband in a house in the countryside. She drives occasionally to visit her son's grave who passed away in an accident 5 years ago. She would like to resume painting, but she feels quite tired. |

*Precisions: IMAD (Institution Genevoise de Maintien à Domicile) - an organization that provides medical and social services to elderly or disabled individuals in their homes ; EMS (Etablissement medico-social) - equivalent to nursing home ; HUG (Hôpitaux universitaires de Genève) - Geneva University hospital ; IEPA(Immeuble avec encadrement pour personnes âgées)/D2/appartement protégé - residence with supervision for older people. Names were changed to preserve anonymity.
